# Race, Gene Expression Signatures, and Clinical Outcomes of Patients With High-Risk Early Breast Cancer

**DOI:** 10.1001/jamanetworkopen.2023.49646

**Published:** 2023-12-28

**Authors:** Beverly Kyalwazi, Christina Yau, Michael J. Campbell, Toshio F. Yoshimatsu, A. Jo Chien, Anne M. Wallace, Andres Forero-Torres, Lajos Pusztai, Erin D. Ellis, Kathy S. Albain, Anne H. Blaes, Barbara B. Haley, Judy C. Boughey, Anthony D. Elias, Amy S. Clark, Claudine J. Isaacs, Rita Nanda, Hyo S. Han, Rachel L. Yung, Debasish Tripathy, Kristen K. Edmiston, Rebecca K. Viscusi, Donald W. Northfelt, Qamar J. Khan, Smita M. Asare, Amy Wilson, Gillian L. Hirst, Ruixiao Lu, William Fraser Symmans, Douglas Yee, Angela M. DeMichele, Laura J. van ’t Veer, Laura J. Esserman, Olufunmilayo I. Olopade

**Affiliations:** 1 Center for Clinical Cancer Genetics and Global Health, The University of Chicago, Chicago, Illinois; 2 Department of Internal Medicine, University of Texas Southwestern Medical Center, Dallas; 3 Department of Surgery, University of California, San Francisco; 4 Department of Medicine, Section of Hematology/Oncology, The University of Chicago, Chicago, Illinois; 5 Department of Hematology Oncology and Surgery, University of California, San Francisco Helen Diller Comprehensive Cancer Center, San Francisco; 6 Division of Breast Surgery and the Comprehensive Breast Health Center, University of California San Diego, La Jolla; 7 Division of Hematology/Oncology, The University of Alabama at Birmingham; 8 Department of Medical Oncology, Yale School of Medicine, Yale University, New Haven, Connecticut; 9 Swedish Cancer Institute, Seattle, Washington; 10 Division of Hematology-Oncology, Department of Medicine, University of Minnesota, Minneapolis; 11 Division of Hematology-Oncology, University of Texas Southwestern Medical Center, Dallas; 12 Department of Surgery, Mayo Clinic, Rochester, Minnesota; 13 University of Colorado Cancer Center, Aurora; 14 Division of Hematology and Oncology, Department of Medicine, University of Pennsylvania, Perelman School of Medicine, Philadelphia; 15 Department of Medicine, Georgetown University, Washington, DC; 16 Department of Breast Oncology, Moffitt Cancer Center, Tampa, Florida; 17 Department of Medicine, School of Medicine, University of Washington, Seattle; 18 Division of Cancer Medicine, Department of Breast Medical Oncology, The University of Texas MD Anderson Cancer Center, Houston; 19 Inova Health System, Fairfax, Virginia; 20 Department of Surgery, University of Arizona College of Medicine, Tucson; 21 Department of Medical Oncology, Mayo Clinic, Phoenix, Arizona; 22 Division of Medical Oncology, Department of Internal Medicine, University of Kansas Medical Center, Kansas City; 23 Quantum Leap Healthcare Collaborative, San Francisco, California; 24 Division of Pathology and Laboratory Medicine, Department of Pathology, The University of Texas MD Anderson Cancer Center, Houston; 25 Department of Laboratory Medicine, University of California, San Francisco Helen Diller Family Comprehensive Cancer Center, San Francisco

## Abstract

**Importance:**

There has been little consideration of genomic risk of recurrence by breast cancer subtype despite evidence of racial disparities in breast cancer outcomes.

**Objective:**

To evaluate associations between clinical trial end points, namely pathologic complete response (pCR) and distant recurrence–free survival (DRFS), and race and examine whether gene expression signatures are associated with outcomes by race.

**Design, Setting, and Participants:**

This retrospective cohort study used data from the Investigation of Serial Studies to Predict Your Therapeutic Response With Imaging and Molecular Analysis 2 (I-SPY 2) multicenter clinical trial of neoadjuvant chemotherapy with novel agents and combinations for patients with previously untreated stage II/III breast cancer. Analyses were conducted of associations between race and short- and long-term outcomes, overall and by receptor subtypes, and their association with 28 expression biomarkers. The trial enrolled 990 female patients between March 30, 2010, and November 5, 2016, with a primary tumor size of 2.5 cm or greater and clinical or molecular high risk based on MammaPrint or hormone receptor (HR)-negative/*ERBB2* (formerly *HER2* or *HER2*/*neu*)–positive subtyping across 9 arms. This data analysis was performed between June 10, 2021, and October 20, 2022.

**Exposure:**

Race, tumor receptor subtypes, and genomic biomarker expression of early breast cancer.

**Main Outcomes and Measures:**

The primary outcomes were pCR and DRFS assessed by race, overall, and by tumor subtype using logistic regression and Cox proportional hazards regression models. The interaction between 28 expression biomarkers and race, considering pCR and DRFS overall and within subtypes, was also evaluated.

**Results:**

The analytic sample included 974 participants (excluding 16 self-reporting as American Indian or Alaska Native, Native Hawaiian or Other Pacific Islander, or multiple races due to small sample sizes), including 68 Asian (7%), 120 Black (12%), and 786 White (81%) patients. Median (range) age at diagnosis was 47 (25-71) years for Asian, 49 (25-77) for Black, and 49 (23-73) years for White patients. The pCR rates were 32% (n = 22) for Asian, 30% for Black (n = 36), and 32% for White (n = 255) patients (*P* = .87). Black patients with HR-positive/*ERBB2*-negative tumors not achieving pCR had significantly worse DRFS than their White counterparts (hazard ratio, 2.28; 95% CI, 1.24-4.21; *P* = .01), with 5-year DRFS rates of 55% (n = 32) and 77% (n = 247), respectively. Black patients with HR-positive/*ERBB2*-negative tumors, compared with White patients, had higher expression of an interferon signature (mean [SD], 0.39 [0.87] and −0.10 [0.99]; *P* = .007) and, compared with Asian patients, had a higher mitotic score (mean [SD], 0.07 [1.08] and −0.69 [1.06]; *P* = .01) and lower estrogen receptor/progesterone receptor signature (mean [SD], 0.31 [0.90] and 1.08 [0.95]; *P* = .008). A transforming growth factor β signature had a significant association with race relative to pCR and DRFS, with a higher signature associated with lower pCR and worse DRFS outcomes among Black patients only.

**Conclusions and Relevance:**

The findings show that women with early high-risk breast cancer who achieve pCR have similarly good outcomes regardless of race, but Black women with HR-positive/*ERBB2*-negative tumors without pCR may have worse DRFS than White women, highlighting the need to develop and test novel biomarker-informed therapies in diverse populations.

## Key Points

**Question:**

Are racial disparities observed in pathologic complete response (pCR) and distant recurrence–free survival (DRFS) in a trial of neoadjuvant chemotherapy (NACT) for patients with breast cancer at high risk of early recurrence?

**Findings:**

In a cohort study of 974 NACT trial patients, no significant differences in pCR or DRFS rates were found among Asian, Black, and White women. Black women with hormone receptor (HR)–positive/*ERBB2* (formerly *HER2* or *HER2/neu*)–negative tumors without pCR had a higher recurrence risk than their White counterparts.

**Meaning:**

These findings suggest that although there is a survival benefit to achieving pCR after NACT across all races, Black women with HR-positive, molecularly high-risk tumors, in the absence of pCR, may fare worse, underscoring the need to better understand heterogeneity in diverse populations.

## Teaser

This cohort study evaluates pathologic complete response and distant recurrence–free survival of early breast cancer by race and whether gene expression signatures correlate with outcomes.

## Introduction

Despite advances in breast cancer treatment with the evolution of immunotherapy and precision oncology, their benefits have not been shared equally. Racial disparities in breast cancer mortality remain a persistent challenge. Black women experience a 40% higher mortality rate than White women.^1^ Such disparities in mortality and clinical outcomes have been attributed to both socioeconomic and genetic risk factors, including limited access to screening and treatment, more advanced-stage breast cancers at the time of diagnosis, and aggressive tumor subtypes observed more often in Black women.^2,3,4,5,6,7^ Despite efforts to identify contributing factors, studies of racial disparities in the clinical trial setting with eyes on differences in tumor biology are limited.^8,9^

The Investigation of Serial Studies to Predict Your Therapeutic Response With Imaging and Molecular Analysis 2 (I-SPY 2) trial is a biomarker-rich, neoadjuvant, adaptively randomized, multicenter, phase 2 platform trial designed for the treatment of locally advanced breast cancer.^10^ Women enrolled in the trial have clinically hormone receptor (HR)–negative/*ERBB2* (formerly *HER2* or *HER2*/*neu*)–positive or genomically (based on molecular subtyping) high-risk breast cancers and are adaptively randomized to different treatment arms based on their tumor subtype. Notably, this trial currently has 26 active sites across the US with approximately 12% of women enrolled identifying as Black or African American. To further investigate racial disparities in treatment outcomes and their potential causes, we performed a comparative analysis of clinical trial outcomes (pathologic complete response [pCR] and distant recurrence–free survival [DRFS]) by race and assessed differences in gene expression signatures among racial groups and their interactions with outcomes.

## Methods

### Study Design

We performed a retrospective cohort analysis of clinical outcomes data by race in the I-SPY 2 trial. The I-SPY 2 uses adaptive randomization to assign patients to control or experimental arms (1:4) based on molecular subtype, as described in prior work.^11,12^ Molecular subtypes were defined by HR status, *ERBB2* status, and risk of recurrence based on a 70-gene assay (MammaPrint; Agendia). Control arm participants received 12 cycles of paclitaxel (in combination with trastuzumab for those with *ERBB2*-positive tumors), followed by 4 cycles of doxorubicin and cyclophosphamide. Experimental arm participants received 1 of 9 experimental agents (neratinib,^11^ veliparib and carboplatin,^12^ trebananib,^13^ ganitumab,^14^ MK-2206,^15^ pertuzumab,^16^ TDM-1 and pertuzumab,^16^ ganetespib,^17^ or a PD-1 inhibitor^18^) in addition to paclitaxel. The primary end point of I-SPY 2 was pCR, defined by the absence of invasive disease in breast and axillary nodes (ypT0/is, ypN0) at the time of surgery. Secondary I-SPY 2 end points were residual cancer burden, event-free survival, and 5-year DRFS. The DRFS was calculated as the time from treatment consent to distant recurrence or death of any cause; patients without events are censored at last known follow-up. All I-SPY 2 participants eligible for analysis had previously signed informed consent for research use of data and specimens. I-SPY 2 was approved by the institutional review boards of all 22 participating sites. The current analysis was approved by the I-SPY 2 Data Access and Publication Committee. This study followed the Strengthening the Reporting of Observational Studies in Epidemiology (STROBE) reporting guideline.^19^

### Participants

The I-SPY 2 cohort consists of 990 women aged 18 years or older with high-risk clinical stage II or III breast cancer and a tumor size of 2.5 cm or larger in diameter who were enrolled between March 30, 2010, and November 5, 2016, at 1 of the 22 clinical sites.^20,21^ Race was self-reported, as collected from case report forms, as American Indian or Alaska Native, Asian, Black, Native Hawaiian or Other Pacific Islander, White, or multiple races. Racial groups with fewer than 10 patients (American Indian or Alaska Native, Native Hawaiian or Other Pacific Islander, and multiple races) were excluded from the analysis. Ethnicity was self-reported as Hispanic or Latino or as not Hispanic or Latino. The present analysis was limited to Asian, Black, and White participants due to the small number of individuals within the other racial groups.

### Gene Expression Analysis

An exploratory, hypothesis-generating analysis was conducted using 28 previously published gene expression biomarkers, including 15 immune cell type–related signatures, 7 immune signaling–related signatures, 1 proliferation signature, estrogen receptor (ER)/progesterone receptor (PR) and *ERBB2* signatures, and mRNA expression of single genes *CD274* (PD-L1), *CD279* (PD-1), and *CD68* (macrophage marker) (eTable 1 in Supplement 1). Signature scores were computed from platform-corrected normalized gene expression data obtained from the National Center for Biotechnology Information Gene Expression Omnibus (GSE194040).^21^ Their association and interaction with race in relation to pCR and DRFS was assessed.

### Statistical Analysis

This data analysis was performed between June 10, 2021, and October 20, 2022. Patient baseline clinical characteristics and demographics were compared using a χ^2^ test for categorical variables and analysis of variance for continuous variables. Logistic regression with significance assessment by the likelihood ratio test was used to assess the association between race and pCR overall and within receptor subtypes. A Cox proportional hazards regression model was used to estimate the hazard ratios and 95% CIs among racial groups (White as reference) overall, within pCR vs non-pCR subsets, and within tumor subtypes by pCR status; significance was assessed using the Wald test. Five-year DRFS among racial groups stratified by pCR status and subtype was estimated using the Kaplan-Meier method. We did not adjust for multiplicities in our analyses within subsets defined by receptor status and pCR. The association between racial groups and expression of 28 gene signatures (related to immune cells, proliferation markers, ER, and *ERBB2* expression) was analyzed using analysis of variance with post hoc Tukey test (using the Tukey-Cramer variation that incorporates adjustments for uneven group sizes) in the overall population and in each receptor subtype without adjustment for multiple hypothesis testing. A 2-sided *P* < .05 was considered statistically significant. Additionally, the interaction between these signatures (dichotomized into the top one-third vs lower two-thirds expression groups) and race in association with pCR and DRFS was assessed using logistic regression and Cox proportional hazards regression models, respectively, with significance assessment using the likelihood ratio test. Analysis was performed using R, version 4.0.2 software (R Project for Statistical Computing).

## Results

### Patient Population

**Figure 1.**
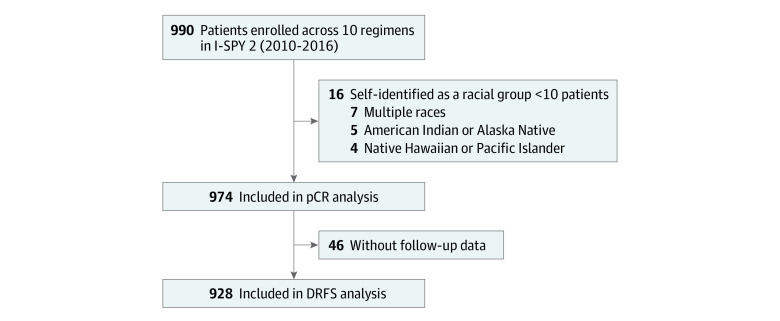
Study Flow Diagram DRFS indicates distant recurrence–free survival; I-SPY 2, Investigation of Serial Studies to Predict Your Therapeutic Response With Imaging and Molecular Analysis 2; pCR, pathologic complete response.

Of the 990 patients in the cohort, 974 were included in the association analysis of the primary end point pCR, with 68 (7%) identifying as Asian, 120 (12%) as Black, and 786 (81%) as White (Figure 1). The median age at diagnosis was similar across racial groups (Asian patients: 47 years [range, 25-71 years]; Black patients: 49 [range, 25-77] years; White patients: 49 years [range, 23-73 years]). The 16 excluded patients were from racial groups with fewer than 10 identified patients. When we compared patient race vs ethnicity, 118 Black patients (98%) identified as non-Hispanic, and 669 White patients (85%) identified as non-Hispanic. No statistically significant differences were observed in patient or tumor characteristics (clinical T and N stage, receptor subtype, and BluePrint molecular subtype) among racial groups (Table 1).

**Table 1.** Patient Demographics

Characteristic	No. (%)			*P* value^a^
	Asian patients (n = 68)	Black patients (n = 120)	White patients (n = 786)	
Ethnicity				
Hispanic or Latino	1 (1)	2 (2)	117 (15)	<.001
Not Hispanic or Latino	67 (99)	118 (98)	669 (85)	
Age, median (range), y	47 (25-71)	49 (25-77)	49 (23-73)	.87
Menopausal status				
Peri or pre	41 (60)	63 (53)	450 (57)	.81
Post	25 (37)	47 (39)	305 (39)	
Unknown	2 (3)	10 (8)	31 (4)	
Longest tumor diameter by MRI, median (range), cm	3.5 (0.4-9.5)	3.7 (1.3-16)	3.7 (0.8-15)	.37
Clinical T stage				
T2^b^	50 (74)	72 (60)	518 (66)	.22
T3/4	15 (22)	40 (33)	238 (30)	
Unknown	3 (4)	8 (7)	30 (4)	
Clinical N status				
LN-negative	37 (54)	46 (38)	361 (46)	.12
LN-positive	27 (40)	64 (53)	384 (49)	
Unknown	4 (6)	10 (8)	41 (5)	
Receptor subtype				
HR-positive/*ERBB2*-negative	21 (31)	44 (37)	310 (39)	.09
HR-negative/*ERBB2*-negative	26 (38)	51 (43)	281 (36)	
HR-positive/*ERBB2*-positive	10 (15)	12 (10)	132 (17)	
HR-negative/*ERBB2*-positive	11 (16)	13 (11)	63 (8)	
BluePrint molecular subtype				
Luminal	22 (32)	33 (28)	266 (34)	.25
Basal	30 (44)	69 (58)	382 (49)	
*ERBB2*	15 (22)	16 (13)	132 (17)	
Unknown	1 (1)	2 (2)	6 (1)	
pCR	22 (32)	36 (30)	255 (32)	.87
Non-pCR^c^	46 (68)	84 (70)	531 (68)	
Residual cancer burden class				
0	22 (32)	36 (30)	261 (33)	.88
1	7 (10)	19 (16)	107 (14)	
2	25 (37)	42 (35)	265 (34)	
3	11 (16)	14 (12)	120 (15)	
Unknown	3 (4)	9 (8)	33 (4)	

Abbreviations: HR, hormone receptor; LN, lymph node; MRI, magnetic resonance imaging; pCR, pathologic complete response.

^a^ *P* value from χ^2^ test for categorical variables and *F* test for continuous variables; unknown values were excluded.

^b^ Includes a small number of patients with T1 tumors who met eligibility criteria by MRI.

^c^ Patients who did not undergo surgery, left their treating institution, or received nonprotocol therapy were considered not to have achieved pCR per protocol.

### Clinical Outcomes by Race

**Figure 2.**
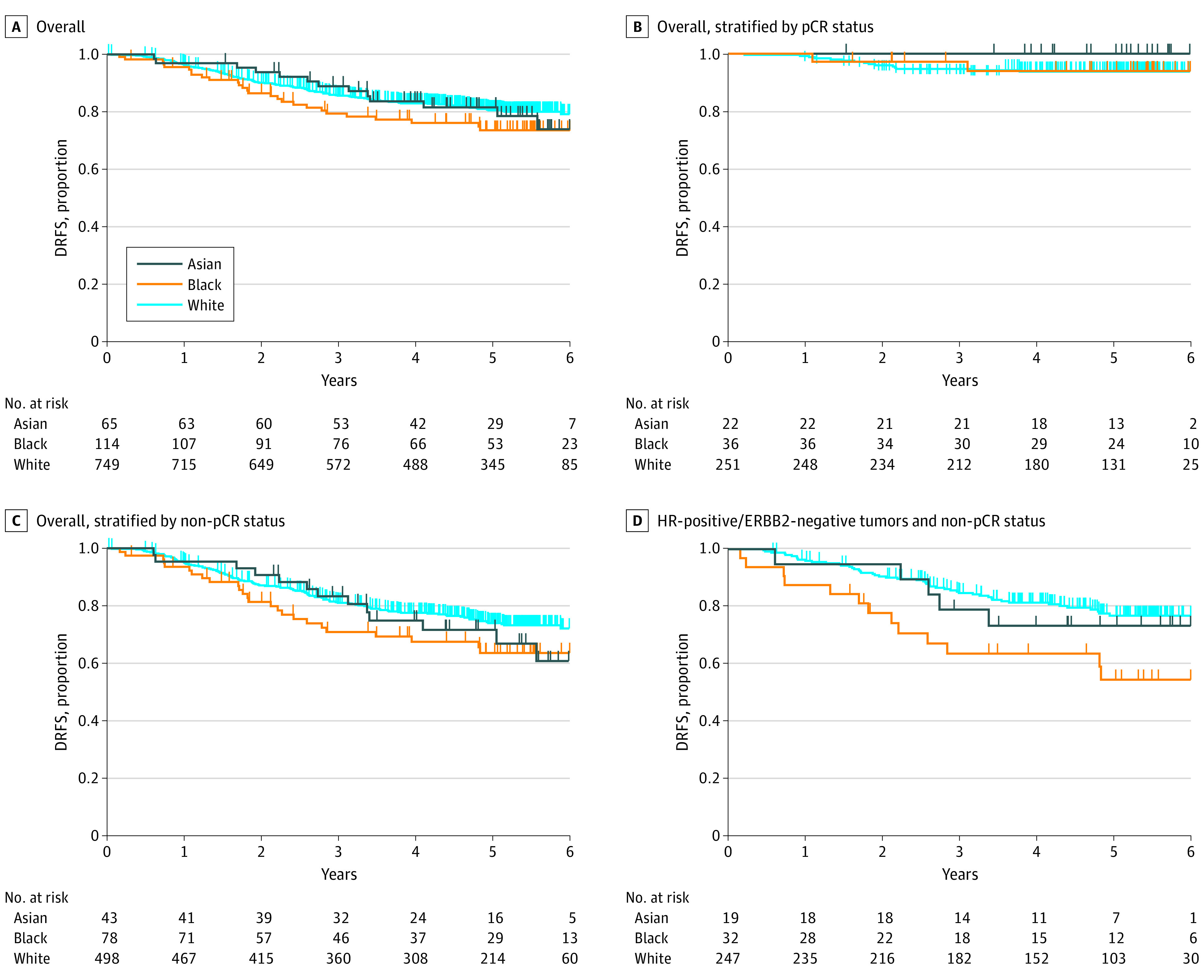
Kaplan-Meier Curves of Distant Recurrence–Free Survival (DRFS) Differences by Race and Pathologic Complete Response (pCR) Status A, Hazard ratios 1.06 (95% CI, 0.60-1.88; *P* = .84) and 1.37 (95% CI, 0.90-2.06; *P* = .14) for Asian and Black patients relative to White patients. B, Hazard ratios 0.00 and 0.93 (95% CI, 0.21-4.07; *P* = .92) for Asian and Black patients relative to White patients. C, Hazard ratios 1.23 (95% CI, 0.69-2.18; *P* = .48) and 1.45 (95% CI, 0.95-2.24; *P* = .09) for Asian and Black patients relative to White patients. D, Hazard ratios 1.26 (95% CI, 0.50-3.17; *P* = .62) and 2.28 (95% CI, 1.24-4.21; *P* = .01) for Asian and Black patients relative to White patients. HR indicates hormone receptor.

Of 974 patients, 313 (32%) achieved pCR. The pCR rate was, 32% for Asian (n = 22), 30% for Black (n = 36), and 32% for White (n = 255) patients (*P* = .87) (Table 2). We found no association between race and pCR among any of the receptor subtypes (Table 2). As of October 28, 2021, follow-up data were available for 928 patients (Figure 1). There were 177 DRFS events, and median follow-up was 5.0 years (range, 0.0-10.2 years). There was no significant difference in DRFS among racial groups, with a hazard ratio of 1.37 (95% CI, 0.90-2.06) and 1.06 (95% CI, 0.60-1.88) between Black and Asian patients, respectively, relative to White patients (Figure 2A). No significant DRFS differences were observed among racial groups within patient subsets stratified by pCR status (Figure 2B and C). Within receptor subtype, we observed a significant difference in DRFS by race (hazard ratio, 2.28; 95% CI, 1.24-4.21; *P* = .01), where White patients with HR-positive/*ERBB2*-negative tumors who did not achieve pCR had a 77% 5-year DRFS rate (n = 247) compared with 55% (n = 32) for similar Black patients (Figure 2D; eTable 2 in Supplement 1). No other significant differences in DRFS by racial groups were observed in subgroup analyses among the other tumor receptor subtypes (including triple-negative breast cancer) by pCR status (eTable 2 and eFigure 1 in Supplement 1).

**Table 2.** Clinical Outcomes by Race and Receptor Status

Receptor status	No. of patients	No. (%)		*P* value
		pCR	No pCR	
All				
Asian	68	22 (32)	46 (68)	.87
Black	120	36 (30)	84 (70)	
White	786	255 (32)	531 (68)	
HR-positive/*ERBB2*-negative				
Asian	21	2 (10)	19 (90)	.52
Black	44	9 (20)	35 (80)	
White	310	51 (16)	259 (84)	
HR-negative/*ERBB2*-negative				
Asian	26	11 (42)	15 (58)	.48
Black	51	16 (31)	35 (69)	
White	281	112 (40)	169 (60)	
HR-positive/*ERBB2*-positive				
Asian	10	4 (40)	6 (60)	.65
Black	12	3 (25)	9 (75)	
White	132	50 (38)	82 (62)	
HR-negative/*ERBB2*-positive				
Asian	11	5 (45)	5 (55)	.41
Black	13	8 (62)	5 (38)	
White	63	42 (67)	21 (33)	

Abbreviations: HR, hormone receptor; pCR, pathologic complete response.

### Gene Expression Signatures by Race

**Figure 3.**
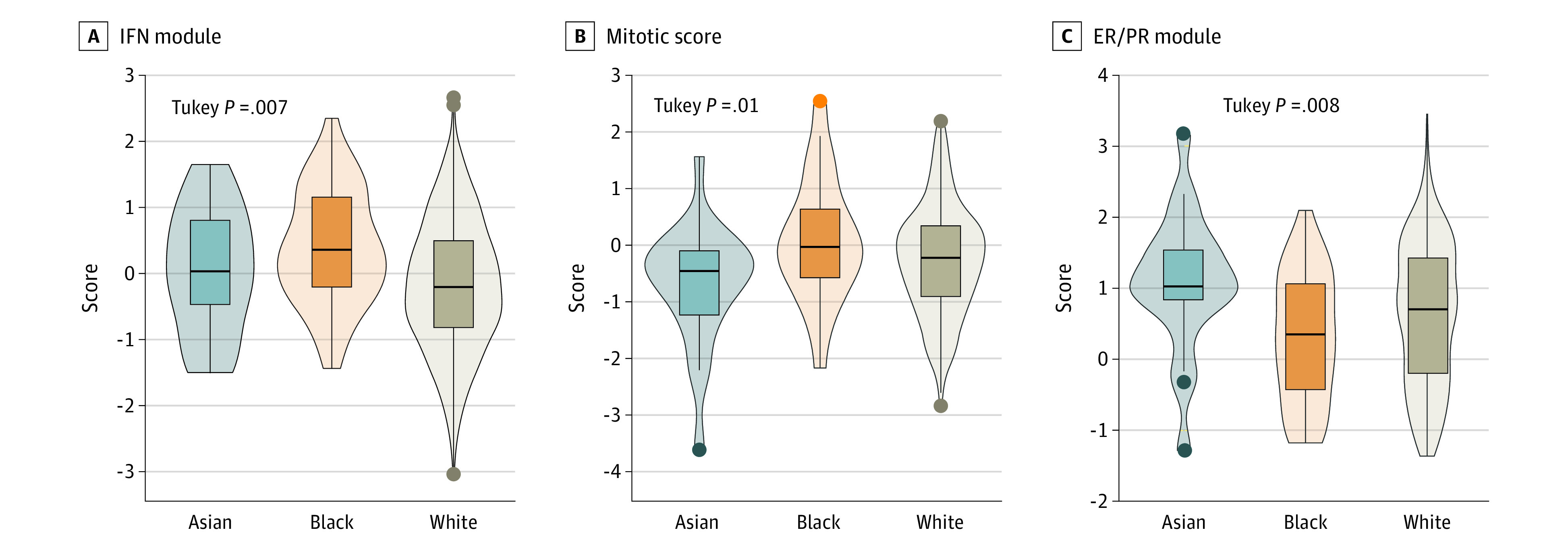
Gene Expression Signatures of Hormone Receptor (HR)–Positive/ERBB-Negative Tumors by Race Boxes indicate the IQR, with the center line indicating the median and whiskers indicating the lower quartile minus 1.5 times the IQR and upper quartile plus 1.5 times the IQR. ER/PR indicates estrogen receptor/progesterone receptor; IFN, interferon.

Among the 28 expression signatures evaluated, 4 were differentially expressed among racial groups within the overall population (*F* test *P* < .05): interferon (IFN) module,^22^ B-cell signature,^23^ dendritic cell signature,^23^ and mitotic score^24^ (eTable 3 in Supplement 2). Among patients with HR-positive/*ERBB2*-negative tumors, 3 signatures (IFN module, mitotic score, and ER/PR module) were differentially expressed among the racial groups (Figure 3A-C; eTable 3 in Supplement 2). Black patients, compared with White patients, had significantly higher expression of the IFN module signature (mean [SD], 0.39 [0.87] and −0.10 [0.99]; *P* = .007); Black patients had a significantly higher expression of mitotic score signature (mean [SD], 0.07 [1.08] and −0.69 [1.06]; *P* = .01) and a lower expression of ER/PR module signature (mean [SD], 0.31 [0.90] and 1.08 [0.95]; *P* = .008) than Asian patients. While higher expression levels of both IFN module and mitotic score signatures were not associated with worse survival outcomes among patients with HR-positive/*ERBB2*-negative tumors (eFigure 2 in Supplement 1), higher expression of the ER/PR module signature was associated with better survival outcomes (hazard ratio, 0.77; 0.60-0.98; *P* = .03). Among the 28 signatures, only the transforming growth factor β (TGF-β) signature^25^ had a significant interaction with race relative to pCR (ratio of ORs associated with TGF-β expression between Black and White patients, 0.32; 95% CI, 0.11-0.84; *P* = .04) and DRFS outcomes (ratio of HRs, 2.73; 95% CI, 1.16-6.41; *P* = .02) when we dichotomized the population by expression of the TGF-β signature (top one-third vs lower two-thirds) (eTable 4 in Supplement 2). While higher or lower expression of TGF-β was not associated with pCR or DRFS outcomes in White and Asian patients, Black patients with a higher TGF-β signature had significantly worse pCR and DRFS outcomes (pCR rate, 7 of 43 vs 29 of 77 [χ^2^ *P* = .02]; high relative to low group: HR, 3.22 [95% CI, 1.47-7.04; log-rank *P* = .002]) (eFigure 2 in Supplement 1).

## Discussion

In this retrospective cohort study, we compared clinical outcomes in the I-SPY 2 trial across patient racial groups among women with clinically (*ERBB2*-positive or HR-negative) or genomically (based on MammaPrint molecular subtyping) high-risk breast cancer. Our findings suggest that there is no association between race and pCR when patients have early access to clinical trials. Consistent with findings that pCR is strongly associated with event-free survival and DRFS,^20^ our analysis supports that women with high-risk breast cancers who receive biomarker-informed neoadjuvant chemotherapy (NACT) and achieve pCR may experience a survival benefit independent of their self-identified race.

### Residual Disease in HR-Positive/*ERBB2*-Negative Subtypes and Differences in DRFS by Race

Strikingly, we found that among women who did not achieve pCR, statistically significant differences in DRFS were observed only among women with HR-positive/*ERBB2*-negative tumors. Within this subtype, Black women experience more than double the risk of recurrence compared with White women. This finding supports the growing literature on racial disparities in breast cancer outcomes, particularly among women with HR-positive/*ERBB2*-negative tumors, and warrants further investigation into the heterogeneity in the biology within this receptor subtype to elucidate this disparity.^5,26,27,28,29,30,31,32^ Recent results from a 690-patient, single-institution study at The University of Chicago, replicated in the larger National Cancer Database, suggested that tumor grade may be the factor accounting most for racial disparities in overall survival among women with HR-positive/*ERBB2*-negative tumors.^33,34^ These findings align with our observations of racial disparities in survival among women with high-risk HR-positive/*ERBB2*-negative tumors. Interestingly, despite that Black women have higher rates of tiple-negative breast cancer,^2^ we did not observe significant racial disparities in outcomes within this subtype of nonresponders.

With the advent of breast cancer molecular subtyping, treatment guidance regarding who may benefit from chemotherapy has evolved.^35,36,37^ Several studies have assessed racial disparities in clinical outcomes for women with ER-positive breast cancers using the 21-gene Oncotype DX Breast Recurrence Score Test. Albain et al^26^ evaluated data from the randomized Trial Assigning Individualized Options for Treatment (TAILORx) that included 9719 patients, of whom 693 were Black women with HR-positive/*ERBB2*-negative, axillary node–negative breast cancer. The investigators found that among women with intermediate recurrence risk based on recurrence scores (RSs), Black women had higher recurrence and mortality rates than White women after adjusting for RS and other comorbidities. A retrospective cohort study of patients with ER-positive breast cancer using the Surveillance, Epidemiology, and End Results Oncotype DX database and the same RS categorizations from the TAILORx study showed that the mortality disparity between Black women (increased mortality) compared with White women persisted in all RS risk groups (low-risk group [RS 0-10]: subdistribution hazard ratio, 2.54 [95% CI, 1.44-4.50]; intermediate-risk group [RS 11-25]: 1.64 [95% CI, 1.23-2.18]; high-risk group [RS >25]: 1.48 [95% CI, 1.10-1.98]).^27^ Although RS is associated with breast cancer–specific mortality in both racial groups, it has been shown to have less prognostic value for Black women than for White women, which may be in part because it was developed in a predominantly non-Hispanic White population.^38,39^ However, these studies are limited to an analysis of clinical outcome differences among women with ER-positive breast cancer. In our study, we used data from the I-SPY 2 clinical trial to look at racial differences in clinical outcomes across multiple receptor subtypes among women considered to have high-risk breast cancers by MammaPrint subtyping.

### Exploratory Analysis of Gene Expression Signatures

Our exploratory analysis of gene expression signatures among women with HR-positive/*ERBB2*-negative tumors revealed 3 differentially expressed gene signatures (IFN module, mitotic score, and ER/PR module) by race. Higher expression of the ER/PR module was associated with better outcomes for patients with the HR-positive/*ERBB2*-negative subtype. Lower expression of this signature among Black compared with Asian patients may have implications when it comes to response to endocrine therapies among patients with this subtype. Though these findings are preliminary, they suggest a pathway for further study of racial disparities in pCR and DRFS among women with HR-positive/*ERBB2*-negative tumors.

A study with molecular methods used in a report by Byun et al^40^ suggested that differential expression of regulatory genes may account for some differences in clinical outcomes that are associated with race among homogeneous tumor receptor groups. In prior reports describing the evolutionary trajectory of breast cancer in the Nigerian Breast Cancer Study and The Cancer Genome Atlas, Black patients with HR-positive/*ERBB2*-negative tumors have higher rates of genomic instability, increased intratumoral heterogeneity, and higher rates of *GATA3* variations, with implications for precision therapeutics among populations of African ancestry.^41,42^ Additional studies that include data from gene expression profiling and assessment of the tumor immune microenvironment of HR-positive/*ERBB2*-negative tumors^43,44^ are promising avenues toward insight into the heterogeneity of this tumor subtype and consequential racial disparities in the survival outcomes observed.

Leveraging the expression data across all subtypes, we found a significant interaction between the TGF-β signature and pCR and DRFS outcomes among racial groups. This finding is remarkable given previous studies on the association between TGF-β signaling and racial disparities in prostate cancer.^45,46^ These studies suggest that higher TGF-β signaling may be associated with more aggressive prostate cancer in Black patients.^38,39^ In early breast cancer, we observed higher levels of expression of the TGF-β signature among Black patients that were associated with lower pCR and DRFS rates, where no association existed for Asian and White patients.

### Strengths and Limitations

Unique strengths of our analysis include a predefined population with a uniformly high risk for breast cancer recurrence; subgroup analyses that accounted for receptor subtype differences; and the use of data from a robust, adaptive clinical trial. Additionally, I-SPY 2 is a multicenter clinical trial that includes a diverse population. Previous reports on racial disparities in breast cancer survival have been observed for reasons that are poorly understood in part due to low enrollment of women who self-identify as part of a racial minority group.^47,48,49^ The percentage of Black women enrolled in the I-SPY 2 trial is 12%, which is proportional to the population of non-Hispanic Black individuals in the US (12.1% of the total US population of 331.9 million as of 2021),^50^ reflecting an intentional selection of clinical sites in geographic areas with diverse populations and improving our ability to analyze clinical outcomes by race. While it has been an aspiration to implement this practice in the general clinical trial setting to reduce breast cancer disparities, more work needs to be done.^51,52^

This study also had several limitations. Lack of sociodemographic and comorbidity data limited our ability to account for social determinants that contribute to racial disparities in clinical outcomes. However, prior studies examining socioeconomic factors contributing to the mortality disparity in breast cancer have found that when adjusting for indicators of social determinants of health (insurance and neighborhood deprivation), the association with self-reported race and disparity in outcomes is persistent.^6,53^ Although our study includes more than 900 patients, the considerable heterogeneity in the pattern of gene expression profiles among relatively smaller-sized subsets of race and breast cancer subtypes limits our ability to determine both statistically and clinically meaningful differences in outcomes by race. Further studies are underway, with additional investigation into biomarkers associated with worse outcomes to inform how we think about potential therapeutic targets and their possible contribution to reducing racial disparities in clinical outcomes.

## Conclusions

In this retrospective cohort study of I-SPY 2 clinical outcomes data, no significant association was found between race and pCR. We conclude that when women with high-risk breast cancer are enrolled in biomarker-informed NACT trials, their survival outcomes can be estimated by achievement of pCR, regardless of race. Our findings reveal evidence of racial disparities in DRFS among women with early-stage, molecularly high-risk, HR-positive/*ERBB2*-negative tumors who do not achieve pCR, with Black women having a significantly higher recurrence risk than White women in this subgroup. This finding suggests a need for further investigation into the heterogeneity of HR-positive/*ERBB2*-negative tumors using gene expression analysis and into the tumor immune microenvironment to provide insight into racial disparities in breast cancer clinical outcomes. Ultimately, our findings underscore the importance of enrolling diverse patient populations in clinical trials to work toward advancing health equity and to better understand contributors to racial disparities in breast cancer mortality.

## Notes

Supplement 1. **eFigure 1.** DRFS Hazard Ratios **eFigure 2.** Associations of Gene Expression Signatures by Race **eTable 1.** Expression Biomarkers Evaluated **eTable 2.** Five-Year Distance Recurrence–Free Survival by Race, pCR Status, and Receptor Subtypes
eReferences

Supplement 2. **eTable 3.** Association Between Expression Signatures Evaluated and Race, Overall and by Tumor Subtypes **eTable 4.** Interaction Between High- vs Low-Signature Expression, Race, and Outcomes (Overall Population)

Supplement 3.
Data Sharing Statement
