# Supplementary material for: Race, Gene Expression Signatures, and Clinical Outcomes of Patients With High-Risk Early Breast Cancer
Source: JAMA Netw Open. 2023 Dec 28;6(12):e2349646. doi: 10.1001/jamanetworkopen.2023.49646 (PMC10755617; doi:10.1001/jamanetworkopen.2023.49646)
Supplement: Supplement 1. — eFigure 1. DRFS Hazard Ratios eFigure 2. Associations of Gene Expression Signatures by Race eTable 1. Expression Biomarkers Evaluated eTable 2. Five-Year Distance Recurrence–Free Survival by Race, pCR Status, and Receptor Subtypes eReferences [file jamanetwopen-e2349646-s001.pdf]

Supplemental Online Content

Kyalwazi B, Yau C, Campbell ML, et al. Race, gene expression signatures, and clinical outcomes among patients with early breast cancer. *JAMA Netw Open*. 2023;6(12):e2349646. doi:10.1001/jamanetworkopen.2023.49646

eFigure 1. DRFS Hazard Ratios

eFigure 2. Associations of Gene Expression Signatures by Race

eTable 1. Expression Biomarkers Evaluated

eTable 2. Five-Year Distance Recurrence–Free Survival by Race, pCR Status, and Receptor Subtypes

eReferences

This supplemental material has been provided by the authors to give readers additional information about their work.

© 2023 Kyalwazi B, et al. *JAMA Network Open.*

eFigure 1. DRFS Hazard Ratios

Estimated DRFS hazard ratios and 90% confidence interval by Cox proportional hazards model among racial groups (White patients as reference) overall, within pCR vs. non-pCR subsets as well as within subtypes by pCR status

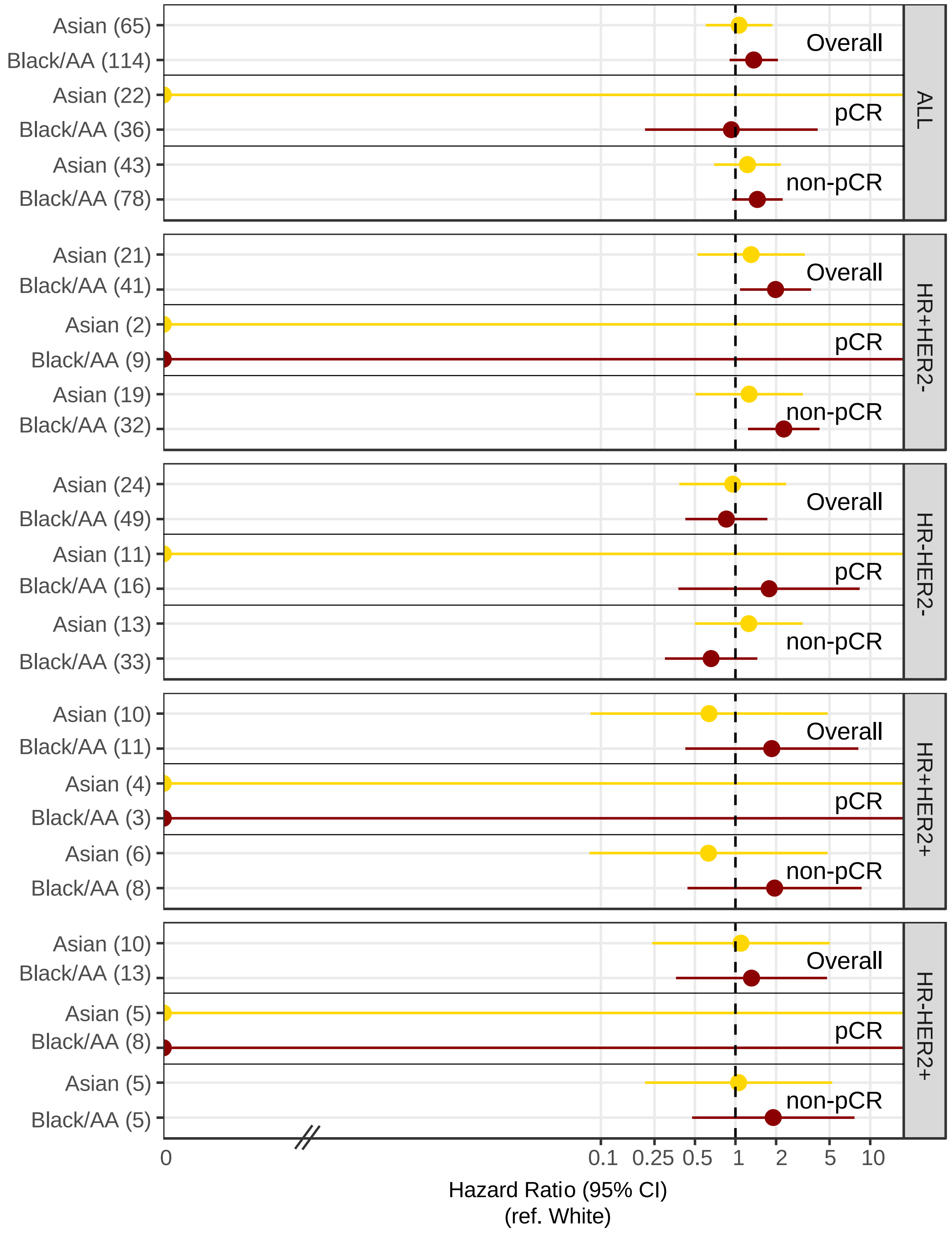

© 2023 Kyalwazi B, et al. *JAMA Network Open.*

eFigure 2. Associations of Gene Expression Signatures by Race.
(A) Association of IFN module, Mitotic score, and ER/PR module to survival outcomes among patients with HR-positive/HER2-negative tumors. Interaction of TGFβ signature with race in relationship to (B) pCR and (C) DRFS outcomes.

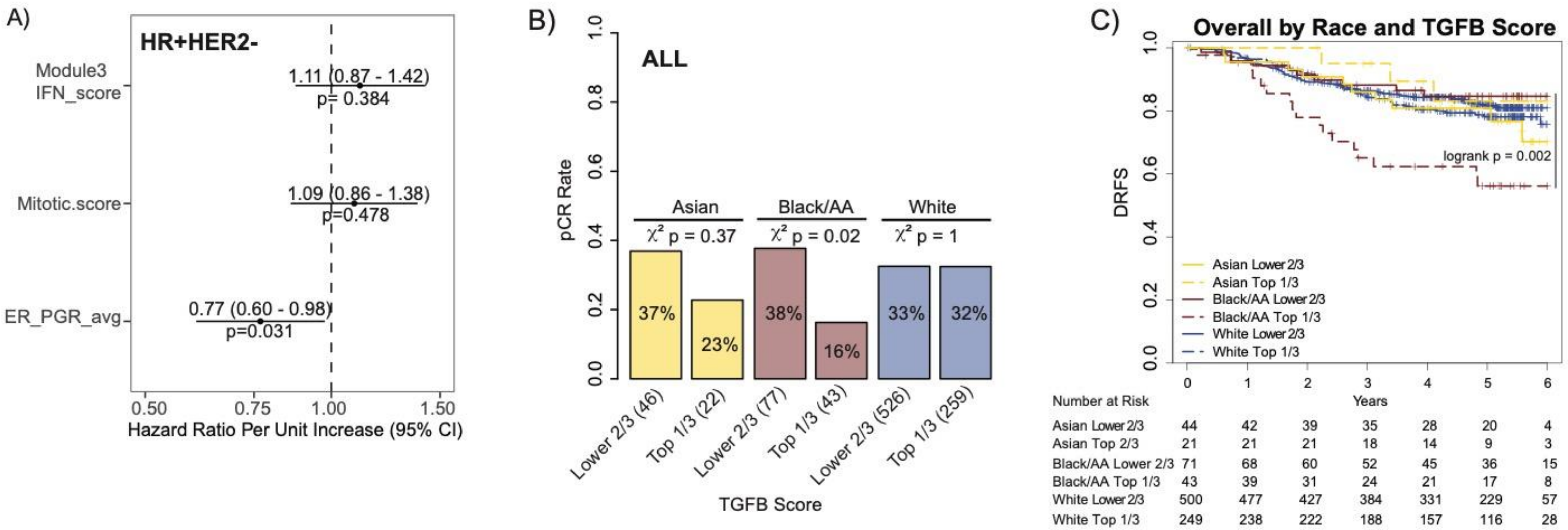

© 2023 Kyalwazi B, et al. *JAMA Network Open.*

eTable 1. Expression Biomarkers Evaluated

Biomarker	Type	Description	Genes	Scoring method	Source
TIL_sig	Immune cell population	Tumor infiltrating lymphocytes signature	PTPRC	1) Mean center, 2) Z-score	Danaher et al.[1]
Tcell_sig	Immune cell population	T cells signature	CD3D, CD3E, CD3G, CD6, SH2D1A, TRAT1	1) Average over genes, 2) mean center, 3) Z-score	Danaher et al.[1]
Tc_sig	Immune cell population	cytotoxic T cells signature	CD8A, CD8B	1) Average over genes, 2) mean center, 3) Z-score	Danaher et al.[1]
ExhTc_sig	Immune cell population	exhausted Tc cells signature	CD244, EOMES, LAG3, PTGER4	1) Average over genes, 2) mean center, 3) Z-score	Danaher et al.[1]
Th1_sig	Immune cell population	type 1 helper T cells signature	TBX21	1) Mean center, 2) Z-score	Danaher et al.[1]
Treg_sig	Immune cell population	regulatory T cells signature	FOXP3	1) Mean center, 2) Z-score	Danaher et al.[1]
Cyto_sig	Immune cell population	cytotoxic cells signature	CTSW, GNLY, GZMA, GZMB, GZMH, KLRB1, KLRD1, KLRK1, NKG7, PRF1	1) Average over genes, 2) mean center, 3) Z-score	Danaher et al.[1]
NK_sig	Immune cell population	Natural killer cells signature	NCR1, XCL1, XCL2	1) Average over genes, 2) mean center, 3) Z-score	Danaher et al.[1]
NK56d_sig	Immune cell population	CD56dim natural killer cells signature	IL21R, KIR3DL1, KIR3DL2	1) Average over genes, 2) mean center, 3) Z-score	Danaher et al.[1]
Bcell_sig	Immune cell population	B cells signature	BLK, CD19, FCRL2, KIAA0125, MS4A1, PNOC, SPIB, TCL1A, TNFRSF17	1) Average over genes, 2) mean center, 3) Z-score	Danaher et al.[1]
DC_sig	Immune cell population	Dendritic cells signature	CCL13, CD209, HSD11B1	1) Average over genes, 2) mean center, 3) Z-score	Danaher et al.[1]
Mac_sig	Immune cell population	Macrophages signature	CD163, CD68, CD84, MS4A4A	1) Average over genes, 2) mean center, 3) Z-score	Danaher et al.[1]
Neut_sig	Immune cell population	Neutrophils signature	CEACAM3, CSF3R, FCAR, FCGR3B, FPR1, S100A12, SIGLEC5	1) Average over genes, 2) mean center, 3) Z-score	Danaher et al.[1]
Mast_sig	Immune cell population	Mast cells signature	CPA3, HDC, MS4A2, TPSAB1, TPSB2	1) Mean center, 2) average over genes, 3) Z-score	Danaher et al.[1]
Mod4_TB	Immune cell population	T and B cell immune module	CD96, CD52, SEMA4D, CXCL13, SP140, CCR7, CTSW, DOCK2, EVI2B, FCN1, KLRK1, FLI1, PLCL2, FYB, IPCEF1, PPP1R16B, CCDC69, STAP1, GPR18, ICOS,GPR171, GZMA, GZMB, GZMK, IGJ, IL2RB, IL2RG, IL7R, ITGA4, ITK, KLRB1, LCK, LGALS2, LRMP, LTB, SH2D1A, CXCL9, NCF4, GIMAP6, IL21R, TRAT1, PLAC8, UBASH3A, POU2AF1, RHOF, LAX1, BANK1, SIRPG, PRF1, DOCK10, PRKCB, CRTAM, PTGDS, PTPRC, PTPRCAP, TNFRSF17, CCL19, SELL, BCL11B, SLAMF1, TNFRSF1B, CCR2, TRAF3IP3, TCL1A, VNN2, PSTPIP1, CD2, CD3G, CD247, CD7, CD8A, CD19, MS4A1, CD27, AIM2, CD37, CYTIP, CD69, CD79A, FAM65B, KIAA0125, P2RY14	1) Mean center, 2) take modified inner product with centroid as published and described below (though averaging would yield similar results), 3) Z-score	Wolf et al.[2]
Mod3_IFN	Immune signaling	Interferon module	IFI44, IFI44L, DDX58, IFI6, IFI27, IFIT2, IFIT1, IFIT3, CXCL10, MX1, OAS1, OAS2, OAS3, HERC5, SAMD9, HERC6, DDX60, RTP4, IFIH1, STAT1, TAP1, OASL, RSAD2, ISG15	1) Mean center, 2) take modified inner product with centroid as published and described below (though averaging would yield similar results), 3) Z-score	Wolf et al.[2]
TGFB_sig	Immune signaling	Transforming growth factor b signature	MMP3, MARCKSL1, IGF2R, LAMB1, SPARC, FN1, ITGA4, SMO, MMP19, ITGB8, ITGA5, NID1, TIMP1, SEMA3F, RHOQ, CTNNB1, MMP2, SERPINE1, EPHB2, COL16A1, EPHA2, TNC, JUP, ITGA3, TCF7L2, COL3A1, CDH6, WNT2B, ADAM9, DSP, HSPG2, ARHGAP1, ITGB5, IGFBP5, ARHGDIA, LRP1, IGFBP2, CTNNA1, LRRC17, MMP14, NEO1, EFNA5, ITGB3, EPHB3, CD44, IGFBP4, TNFRSF1A, RAC1, PXN, PLAT, COL8A1, WNT8B, IGFBP3, RHOA, EPHB4, MMP1, PAK1, MTA1, THBS2, CSPG2, MMP17, CD59, DVL3, RHOB, COL6A3, NOTCH2,	1) Mean center, 2) average over genes, 3) Z-score	Teschendorff et al.[3]

© 2023 Kyalwazi B, et al. *JAMA Network Open.*

			BSG, MMP11, COL1A2, ZYX, RND3, THBS1, RHOG, ICAM1, LAMA4, DVL1, PAK2, ITGB2, COL6A1, FGD1,		
STAT1_sig	Immune signaling	signal transducer and activator of transcription 1 signature	TAP1, GBP1, IFIH1, PSMB9, CXCL9, IRF1, CXCL11, CXCL10, IDO1, STAT1	1) Mean center, 2) average over genes, 3) Z-score	Rody et al.[4]
ICS5	Immune signaling	Integrated Cytokine Score	CXCL13, CLIC5, HLA-F, TNFRSF17, XCL2	1) Mean center, 2) average over genes, 3) Z-score	Yau et al.[5]
Chemokine12	Immune signaling	Signature of 12 chemokines	CCL2, CCL3, CCL4, CCL5, CCL8, CCL18, CCL19, CCL21, CXCL9, CXCL10, CXCL11, CXCL13	1) Mean center, 2) average over genes, 3) Z-score	Coppola et al.[6]; Prabhakaran et al.[7]
TIS	Immune signaling	Tumor inflammatory signature	TIGIT, CD27, CD8A, PDCD1LG2, CXCR6, LAG3, CD274, CMKLR1, NKG7, CCL5, PSMB10, ID01, PPBP, HLA-DQA1, CD276, STAT1, HLA-DRB1, HLA-E	1) Mean center, 2) average over genes, 3) Z-score	Ayers et al.[8]
Geparsixto	Immune signaling	GeparSixto TRIAL immune activation signature	CXCL9, CCL5, CD8A, CD80, CXCL13, IDO1, PDCD1, CD274, CTLA4, FOXP3	1) Mean center, 2) average over genes, 3) Z-score	Denkert et al.[9]
ER_PR_sig	hormone receptor	Estrogen and progesterone receptor expression	ESR1, PGR	1) Mean center, 2) average over genes, 3) Z-score	
Mitototic_sig	proliferation	Proliferation/cell cycle signature	PLK1, CDK1, BUB1B, NEK2, TTK, MELK, PLK4, CHEK1, AURKA, AURKB, BUB1, PBK	1) Mean center, 2) average over genes, 3) Z-score	Bianchini et al.[10]
PD1	Immune (single marker)	PD1 gene expression	PDCD1	Z-score	
PDL1	Immune (single marker)	PDL1 gene expression	CD274	Z-score	
CD68	Immune (single marker)	CD68 gene expression	CD68	Z-score	
Module7_ERBB2	ERBB2	ERBB2 co-expression module	ERBB2, GRB7, STARD3, PGAP3	1) Mean center, 2) take modified inner product with centroid as published and described below, 3) Z-score	Wolf et al.[2]

© 2023 Kyalwazi B, et al. *JAMA Network Open.*

eTable 2. Five-Year Distance Recurrence–Free Survival By Race, pCR Status, and Receptor Subtypes

Race	Response	N	DRFS at 5 years	Hazard Ratio (95% CI)	Wald p
**ALL**					
Asian	ALL	65	82%	1.06(0.6-1.88)	0.84
Black/AA	ALL	114	74%	1.37(0.9-2.06)	0.14
White	ALL	749	81%	REF	
Asian	pCR	22	100%	0(0-Inf)	na
Black/AA	pCR	36	94%	0.93(0.21-4.07)	0.92
White	pCR	251	94%	REF	
Asian	non-pCR	43	72%	1.23(0.69-2.18)	0.48
Black/AA	non-pCR	78	64%	1.45(0.95-2.24)	0.09
White	non-pCR	498	74%	REF	
**HR+HER2-**					
Asian	ALL	21	76%	1.31(0.52-3.27)	0.57
Black/AA	ALL	41	64%	1.98(1.08-3.64)	0.03
White	ALL	298	80%	REF	
Asian	pCR	2	100%	0(0-Inf)	na
Black/AA	pCR	9	100%		na
White	pCR	51	96%	REF	
Asian	non-pCR	19	73%	1.26(0.5-3.17)	0.62
Black/AA	non-pCR	32	55%	2.28(1.24-4.21)	0.01
White	non-pCR	247	77%	REF	
**HR-HER2-**					
Asian	ALL	24	80%	0.95(0.38-2.37)	0.92
Black/AA	ALL	49	79%	0.85(0.42-1.72)	0.66
White	ALL	270	78%	REF	
Asian	pCR	11	100%	0(0-Inf)	na
Black/AA	pCR	16	87%	1.78(0.38-8.36)	0.47
White	pCR	111	93%	REF	
Asian	non-pCR	13	58%	1.26(0.5-3.15)	0.63
Black/AA	non-pCR	33	75%	0.66(0.3-1.45)	0.30
White	non-pCR	159	67%	REF	
**HR+HER2+**					
Asian	ALL	10	100%	0.64(0.08-4.83)	0.66
Black/AA	ALL	11	80%	1.86(0.42-8.16)	0.41
White	ALL	124	88%	REF	
Asian	pCR	4	100%	0(0-Inf)	na
Black/AA	pCR	3	100%	0(0-Inf)	na
White	pCR	49	98%	REF	
Asian	non-pCR	6	100%	0.63(0.08-4.82)	0.66
Black/AA	non-pCR	8	71%	1.95(0.44-8.64)	0.38
White	non-pCR	75	82%	REF	
**HR-HER2+**					
Asian	ALL	10	79%	1.1(0.24-5.01)	0.90
Black/AA	ALL	13	77%	1.31(0.36-4.78)	0.68
White	ALL	57	82%	REF	
Asian	pCR	5	100%	0(0-Inf)	na
Black/AA	pCR	8	100%	0(0-Inf)	na
White	pCR	40	90%	REF	
Asian	non-pCR	5	53%	1.05(0.21-5.23)	0.95
Black/AA	non-pCR	5	40%	1.91(0.48-7.66)	0.36
White	non-pCR	17	63%	REF	

© 2023 Kyalwazi B, et al. *JAMA Network Open.*

eReferences

1. Danaher P, Warren S, Dennis L, et al. Gene expression markers of Tumor Infiltrating Leukocytes. *J Immunother Cancer*. 2017;5(1):18. doi:10.1186/s40425-017-0215-8

2. Wolf DM, Lenburg ME, Yau C, Boudreau A, Veer LJ van 't. Gene Co-Expression Modules as Clinically Relevant Hallmarks of Breast Cancer Diversity. *Plos One*. 2014;9(2):e88309. doi:10.1371/journal.pone.0088309

3. Teschendorff AE, Gomez S, Arenas A, et al. Improved prognostic classification of breast cancer defined by antagonistic activation patterns of immune response pathway modules. *Bmc Cancer*. 2010;10(1):604. doi:10.1186/1471-2407-10-604

4. Rody A, Holtrich U, Pusztai L, et al. T-cell metagene predicts a favorable prognosis in estrogen receptor-negative and HER2-positive breast cancers. *Breast Cancer Res Bcr*. 2009;11(2):R15. doi:10.1186/bcr2234

5. Yau C, Sninsky J, Kwok S, et al. An optimized five-gene multi-platform predictor of hormone receptor negative and triple negative breast cancer metastatic risk. *Breast Cancer Res*. 2013;15(5):R103. doi:10.1186/bcr3567

6. Coppola D, Nebozhyn M, Khalil F, et al. Unique Ectopic Lymph Node-Like Structures Present in Human Primary Colorectal Carcinoma Are Identified by Immune Gene Array Profiling. *Am J Pathology*. 2011;179(1):37-45. doi:10.1016/j.ajpath.2011.03.007

7. Prabhakaran S, Rizk VT, Ma Z, et al. Evaluation of invasive breast cancer samples using a 12-chemokine gene expression score: correlation with clinical outcomes. *Breast Cancer Res*. 2017;19(1):71. doi:10.1186/s13058-017-0864-z

8. Ayers M, Lunceford J, Nebozhyn M, et al. IFN-γ–related mRNA profile predicts clinical response to PD-1 blockade. *J Clin Invest*. 2017;127(8):2930-2940. doi:10.1172/jci91190

9. Denkert C, Minckwitz G von, Brase JC, et al. Tumor-Infiltrating Lymphocytes and Response to Neoadjuvant Chemotherapy With or Without Carboplatin in Human Epidermal Growth Factor Receptor 2–Positive and Triple-Negative Primary Breast Cancers. *J Clin Oncol*. 2014;33(9):983-991. doi:10.1200/jco.2014.58.1967

10. Bianchini G, Pusztai L, Karn T, et al. Proliferation and estrogen signaling can distinguish patients at risk for early versus late relapse among estrogen receptor positive breast cancers. *Breast Cancer Res*. 2013;15(5):R86. doi:10.1186/bcr3481

© 2023 Kyalwazi B, et al. *JAMA Network Open.*
