# Supplementary material for: Race, Gene Expression Signatures, and Clinical Outcomes of Patients With High-Risk Early Breast Cancer
Source: JAMA Netw Open. 2023 Dec 28;6(12):e2349646. doi: 10.1001/jamanetworkopen.2023.49646 (PMC10755617; doi:10.1001/jamanetworkopen.2023.49646)
Supplement: Supplement 3. — Data Sharing Statement [file jamanetwopen-e2349646-s003.pdf]

Data Sharing Statement

Kyalwazi. Race, Gene Expression Signatures, and Clinical Outcomes of Patients With High-Risk Early Breast Cancer. *JAMA Netw Open*. Published December 28, 2023. doi:10.1001/jamanetworkopen.2023.49646

Data
Data available: Yes
Data types: Deidentified participant data
How to access data: Signature scores and normalized gene expression data are publicly available from the NCBI Gene Expression Omnibus (GSE194040). Clinical data will be provided upon reasonable request to the corresponding author.
When available: With publication

Supporting Documents
Document types: None

Additional Information
Who can access the data: Signature scores and normalized gene expression data are publicly available from the NCBI Gene Expression Omnibus (GSE194040). Clinical data will be provided upon reasonable request to the corresponding author.
Types of analyses: Signature scores and normalized gene expression data are publicly available from the NCBI Gene Expression Omnibus (GSE194040). Clinical data will be provided upon reasonable request to the corresponding author.
Mechanisms of data availability: Signature scores and normalized gene expression data are publicly available from the NCBI Gene Expression Omnibus (GSE194040). Clinical data will be provided upon reasonable request to the corresponding author.
